# Use of Simulation Modeling to Inform Decision Making for Health Care Systems and Policy in Colorectal Cancer Screening: Protocol for a Systematic Review

**DOI:** 10.2196/16103

**Published:** 2020-05-13

**Authors:** Heather Smith, Peyman Varshoei, Robin Boushey, Craig Kuziemsky

**Affiliations:** 1 Telfer School of Management University of Ottawa Ottawa, ON Canada; 2 Division of Colorectal Surgery The Ottawa Hospital Department of Surgery Ottawa, ON Canada; 3 Office of Research Services MacEwan University Edmonton, AB Canada

**Keywords:** colorectal cancer screening, simulation modeling, decision-making, model validation

## Abstract

**Background:**

Simulation modeling has frequently been used to assess interventions in complex aspects of health care, such as colorectal cancer (CRC) screening, where clinical trials are not feasible. Simulation models provide estimates of outcomes, unintended consequences, and costs of an intervention; thus offering an invaluable decision aid for policy makers and health care leaders. However, the contribution that simulation models have made to policy and health system decisions is unknown.

**Objective:**

This study aims to assess if simulation modeling has supported evidence-informed decision making in CRC screening.

**Methods:**

A preliminary literature search and pilot screening of 100 references were conducted by three independent reviewers to define and refine the inclusion criteria of this systematic review. Using the developed inclusion criteria, a search of the academic and gray literature published between January 1, 2008, and March 1, 2019, will be conducted to identify studies that developed a simulation model focusing on the delivery of CRC screening of average-risk individuals. The three independent reviewers will assess the validation process and the extent to which the study contributed evidence toward informed decision making (both reported and potential). Validation will be assessed based on adherence to the best practice recommendations described by the International Society for Pharmacoeconomics and Outcomes Research-Society for Medical Decision Making (ISPOR-SMDM). Criteria for potential contribution to decision making will be defined as outlined in the internationally recognized Grading of Recommendations Assessment, Development and Evaluation Evidence to Decision (GRADE EtD) framework. These criteria outline information that the health system and policy decision makers should consider when making an evidence-informed decision including an intervention’s resource utilization, cost-effectiveness, impact on health equity, and feasibility. Subgroup analysis of articles based on their GRADE EtD criteria will be conducted to identify methods associated with decision support capacity (ie, participatory, quantitative, or mixed methods).

**Results:**

A database search of the literature yielded 484 references to screen for inclusion in the systematic review. We anticipate that this systematic review will provide an insight into the contribution of simulation modeling methods to informed decision making in CRC screening delivery and discuss methods that may be associated with a stronger impact on decision making. The project was funded in May 2019. Data collection took place from January 2008 to March 2019. Data analysis was completed in November 2019, and are expected to be published in spring 2020.

**Conclusions:**

Our findings will help guide researchers and health care leaders to mobilize the potential for simulation modeling to inform evidence-informed decisions in CRC screening delivery. The methods of this study may also be replicated to assess the utility of simulation modeling in other areas of complex health care decision making.

**International Registered Report Identifier (IRRID):**

DERR1-10.2196/16103

**Trial Registration:**

PROSPERO no. 130823; https://www.crd.york.ac.uk/PROSPERO

## Introduction

The benefits of colorectal cancer (CRC) screening have been well cited including a reduced incidence of CRC, earlier stage of presentation, improved outcomes for patients with detected malignancy, and reduced CRC-associated mortality [1-4]. Screening uses fecal tests, diagnostic imaging, and endoscopic examination to assess the possiblity of CRC occurring among asymptomatic individuals at increased risk of developing CRC. Participation of eligible individuals is voluntary and remains low in many regions across our country [5]. Determining the best screening modality, delivery method, resource allocation, and follow-up for screening is complex because health policy and system decision makers must weigh the cost and benefits of screening, taking into account the sensitivity, specificity, and accessibility of various screening modalities. Furthermore, such interventions often cannot be tested in clinical trials because of multiple environmental, sociocultural, and health system factors that negate the feasibility and safety of a trial [6]. Researchers and decision makers have increasingly relied on simulation models to evaluate interventions in CRC screening [7,8].

A simulation model is a computer-generated representation of a real-world system or process that can be used to analyze the evolving behavior of a system over time, or modified to predict results of a variety of “what-if” scenarios [9]. Simulation models have been applied to a broad range of areas in health care to predict outcomes, unintended consequences, and costs of proposed interventions, thereby offering an invaluable decision aid for policy makers and health care leaders [7,10-12]. The purpose of simulation models is well defined, that is, to provide decision makers with evidence to facilitate decision making; however, the extent to which simulation modeling has fulfilled this purpose in CRC screening is unknown [13]. Simulation modeling has the potential to provide strong evidence for multiple aspects of informed decision making at the policy and health system level, including a proposed intervention’s resource utilization, cost-effectiveness, feasibility, sustainability, potential impact, and acceptance among stakeholders [14,15]. For instance, by simulating what-if scenarios informed by clinical trials and observational data, the model can help to identify the appropriate age to initiate screening, superiority of one screening modality over another, or the most cost-effective frequency of screening a particular population in the long term. However, the extent to which simulation modeling has realized this potential impact in CRC screening is unknown.

For simulation models to be useful for decision makers, models must be sufficiently accurate and valid for application [13]. There have been concerns with model credibility in health care and reporting of model conceptualization, parameterization, and validation is not consistent in the literature [16-18]. For this reason, in this systematic review, each study will be assessed for adherence to best practice recommendations in model validation as detailed in the Methods section of this proposal [16].

From our review of the literature, we identified two gaps that we plan to address with this systematic review: (1) no systematic review has examined the application of simulation in CRC screening within the last 10 years and (2) no systematic review has specifically addressed the impact of simulation modeling on decision making in health care. The most recent systematic review that had examined CRC screening only included articles until 2007 inclusively [19]. Since that time, systematic reviews have been conducted examining the quality and cost-effectiveness of simulation modeling in breast cancer screening, but not for CRC screening [20,21]. For instance, a systematic review by Sobolev and colleagues [22] looked at the reported “utility” of simulation models in surgical patient flow and reinforced the need for evaluating the impact of models on decision making, but did not formally evaluate this in their review. Therefore, this study aims to address these knowledge gaps by assessing the validity and impact of simulation modeling on health system and policy decision making in CRC screening delivery. Publication of this protocol will allow for critical peer review of the aims and methods outlined for the intended study. This will help strengthen the rigor by which it will be conducted and validate its utility.

## Methods

### Protocol and Registration

This systematic review will be conducted in accordance with the Cochrane Library systematic reviews guide and the Preferred Reporting Items for Systematic reviews and Meta-Analysis (PRISMA) [23,24]. This protocol is reported in accordance with the PRISMA-Protocol checklist (Multimedia Appendix 1). The protocol has been submitted for registration in PROSPERO, and any amendments will be filed with PROSPERO (no. 130823).

### Search Strategy and Eligibility Criteria

Studies will be selected by searching the academic and gray literature published between January 1, 2008, and March 1, 2019, conducted to identify articles that include (1) simulation modeling methods and (2) a focus on CRC screening. Only full-text articles available in English will be included. Studies will be identified from academic databases (Medline, Embase, Cochrane Central, Scopus, Web of Science, IEEEXplore, ACM Digital Library, Econolit, National Health Service Economic Evaluation Database, Health Assessment Database, and Cost-Effective Analysis Registry) using controlled vocabulary (Medical Subject Heading) and text word search terms selected by the author HS and an experienced librarian Alexandra Davis (Textbox 1).

Terms were selected to capture the most commonly used types of simulation models in health care (discrete event simulation, Markov chain model, Monte-Carlo simulation, agent-based model, and system dynamics model) [11]. This will be supplemented by hand searching of the gray literature and conference proceedings as well as citation searches of selected articles.

**Preliminary search strategies.**

Database: Ovid MEDLINE(R) ALL <January 1, 2008 to March 1, 2019> Search Strategy:

1. Systems Analysis/
2. systems thinking.tw,kw.
3. systems science.tw,kw.
4. systems approach.tw,kw.
5. systems theory.tw,kw.
6. systems analysis.tw,kw.
7. system* model*.tw,kw.
8. simulation model*.tw,kw.
9. monte carlo method/ or Markov Chains/
10. (markov or monte carlo).tw,kw.
11. discrete event.tw,kw.
12. agent-based model*.tw,kw.
13. or/1-12
14. (system* dynamics or dynamic systems).tw,kw.
15. colonoscopy/ or sigmoidoscopy/
16. (colonoscop* or sigmoidoscop*).tw,kw.
17. FECES/ch or (f?ecal occult blood test or f?ecal immunochemical test or FOBT or stool DNA or stool test).tw,kw.
18. or/15-17
19. Mass Screening/ or “Early Detection of Cancer”/
20. (screening or “early detection”).tw,kw.
21. 19 or 20
22. exp Colorectal Neoplasms/
23. ((colorectal or colo-rectal or colon* or rectal) adj2 (cancer or neoplasm*)).tw.
24. 22 or 23
25. 21 and 24
26. CRC screen*.tw,kw.
27. 18 or 25 or 26
28. 13 and 27

**Figure 1:**
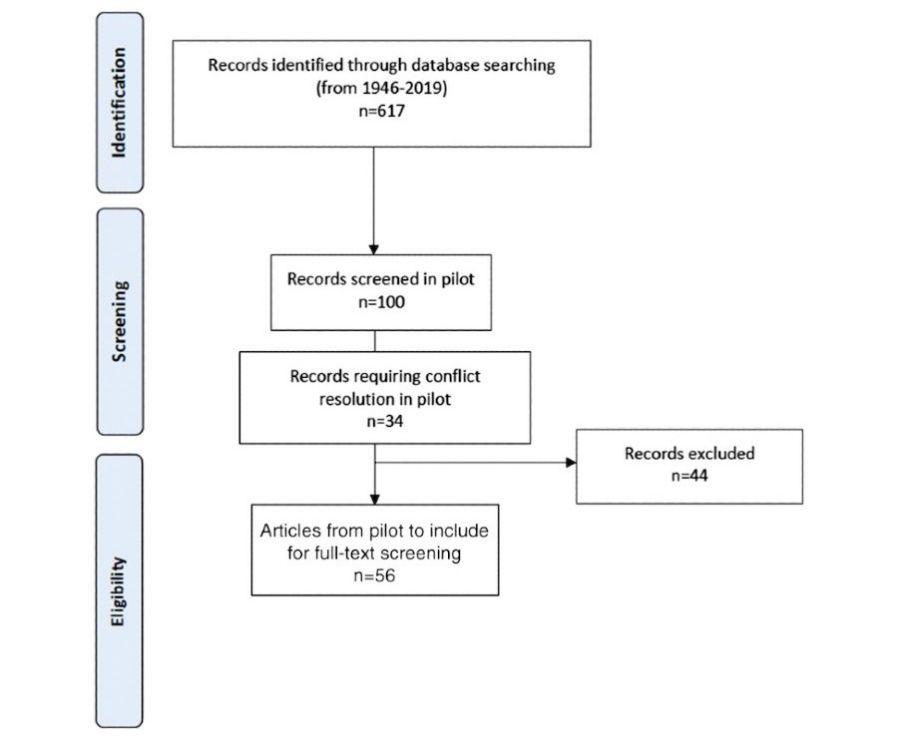
Preferred Reporting Items for Systematic reviews and Meta-Analysis (PRISMA) flow diagram of pilot search and reference screening.

Simulation modeling can be used in a broad scope of applications in cancer screening. To help refine the appropriate inclusion criteria and feasibility of this review, a preliminary search of the literature was conducted. A search of the literature published between January 1, 1946, and March 1, 2019, was conducted, and a pilot screening of 100 abstracts was performed by three independent reviewers (HS, PV, and CK) using the following inclusion criteria: (1) simulation model use and (2) CRC screening. This yielded 56 of 100 included abstracts after 24 conflicts were resolved through extensive discussion among all authors (Figure 1). To reduce conflicts, the inclusion and exclusion criteria were further refined to include only original articles describing a simulation model derived from clinical data focused on the delivery of CRC screening individuals with average CRC risk using one or all of the following modalities of screening recommended by the Canadian guidelines within the last 10 years: fecal occult blood test, fecal immunohistochemical testing, flexible sigmoidoscopy, and colonoscopy [25-27]. Excluded articles are those describing other screening modalities not recommended in the Canadian screening guidelines as identified above, commentary or review articles, simulation models that include screening of other cancers, or articles that have no mention of screening delivery. The time frame was also further restricted to only include articles published after 2008 because a systematic review on the use of simulation modeling in health care, including CRC screening, was identified and had included articles published until the end of 2007 [19]. A second pilot screening was conducted using the revised criteria, which yielded fewer conflicts, and the revised inclusion and exclusion criteria were adopted for this systematic review.

### Selection of Studies for the Review

All article titles and abstracts will be screened by three independent reviewers (HS, PV, and CK) using the abstract screening program Abstrakr (Brown University) followed by screening of selected full-text studies for compliance with the eligibility criteria as mentioned above using DistillerSR, version 2019 (Evidence Partners) [28,29]. Articles or reports of the same study will be linked together. Authors of the articles will be contacted to clarify study eligibility, where appropriate. Conflicts will be resolved through discussion between reviewers or consultation with author (RB), as needed.

### Data Extraction

All included studies will be reviewed by three independent reviewers (HS, CK, and PV). Using DistillerSR version 2019, data will be extracted regarding the study and model description, and model validation as outlined in Table 1 [13]. Discrepancies will be identified and resolved through discussion, and missing data will be requested from the study authors as needed.

The validation of a simulation model is an important determinant of the risk of bias and applicability of a simulation model. All models will be assessed in accordance with the guidelines of the International Society for Pharmacoeconomics and Outcomes Research-Society for Medical Decision Making (ISPOR-SMDM) report [13]. From the literature review, several tools were identified to assist model developers and users with model validation [13,16,17,30-33]. The ISPOR-SMDM Taskforce has developed good practice guidelines for modeling in health care including recommendations on conceptualization, parameterization, and validation. Of the validation tools identified in the literature, the taskforce report had the broadest scope and most rigorous development process; therefore, it was selected to guide validation assessment in this review [16]. Authors (HS, CK, and PV) will individually assess whether authors report or conduct face validity (wherein experts evaluate model structure, data sources, assumptions, and results), verification or internal validity (check accuracy of coding), cross validity (comparison of results with other models analyzing the same problem), external validity (comparing model results with real-world results), and predictive validity (comparing model results with prospectively observed events), as outlined in Table 1 [13].

**Table 1:** Model characteristics and validation.

Characteristics		Description
Country		Location of intended application.
Year		Year of publication.
Simulation model type		Type of approach (ie, system dynamics, Monte Carlo, Markov chain model, agent-based model, discrete event).
Intended application(s)		Area of application (ie, forecasting of cost, resource utilization).
Funding sources		Source of financial support of the project, if applicable.
Stakeholders		Identifies model users/decision makers and their role in the modeling.
**Conceptualization**		
	Parameters defined	Defines the model parameters: values used to either define the characteristics of the model or calculate the performance indicators.
	Structure	Demonstration of variables and their relationships (ie, in graphical formation).
**Operationalization**		
	Model duration	Length of time simulated, number of runs, and if the model was terminating or steady state.
	Inputs	List inputs of model.
	Simulated outputs	List outputs of model.
	Observed results	Observed outcomes, if available.
	Data sources	Type of data sources (ie, primary or secondary).
	Limitations	Assumptions and limitations of the model.
	Software	Type of software used to develop the model.
**Validation methods** [13]		
	Face validation	Model structure, data sources, problem formulation, and results are evaluated by people who have clinical expertise.
	Verification/internal validation	Examination of the extent to which mathematical calculations are performed correctly and are consistent with the model’s specifications.
	Cross validation	Examination of the different models that address the same problem and comparison of their results.
	External validation	Comparison of model’s results with actual event data.
	Predictive validation	Comparison of model’s simulated outcomes to similar clinical trial or cohort study.

Studies will then be assessed for the extent to which the study has or could potentially have contributed evidence toward informed decision making. For reported contribution, each article will be searched in its entirety for statements referring to the simulation model results informing decision making. If not clearly stated in the publication, the information will be requested from study authors.

Recognizing that the impact of a simulation model on a specific decision may not be communicated at the time of publication, we plan to also assess the potential contribution a simulation model could have made to evidence-informed decision making based on whether the results align with important factors for making an informed decision [34]. We will assess articles to determine whether they include evidence considered to be important for decisions, as outlined in the internationally recognized Grading of Recommendations Assessment, Development and Evaluation Evidence to Decision (GRADE EtD) framework [14]. The GRADE EtD framework has been developed as part of the Developing and Evaluating Communication strategies to support Informed Decisions and practice based on Evidence (DECIDE) project in collaboration with researchers in the health system and public health internationally. It outlines a set of important factors that decision makers should consider and address with research evidence to guide their decisions in health policy or systems [35]. These criteria include information on an intervention’s resource utilization, cost-effectiveness, impact on health equity, and feasibility (Table 2). We will assess whether the study results apply to the GRADE EtD criteria. Subgroup analysis of articles based on their GRADE EtD criteria will be conducted to identify methods associated with decision support capacity (ie, quantitative or mixed methods).

**Table 2:** GRADE EtD (Grading of Recommendations Assessment, Development and Evaluation Evidence to Decision) criteria of decision making for health system and public health decisions [14].

Criteria	Detailed questions
Is the problem a priority?	Are the consequences of the problem serious (ie, severe or important in terms of the potential benefits or savings)? Is the problem urgent? (Not relevant for coverage decisions.) Is it a recognized priority (eg, based on a political or policy decision)? (Not relevant when an individual patient perspective is taken.)
How substantial are the desirable anticipated effects?	Judgments for each outcome for which there is a desirable effect.
How substantial are the undesirable anticipated effects?	Judgments for each outcome for which there is an undesirable effect.
What is the overall certainty of the evidence of effects?	See GRADE guidance regarding detailed judgments about the quality of evidence or certainty in estimates of effects.
Is there important uncertainty about or variability in how much people value the main outcome?	Is there important uncertainty about how much people value each of the main outcomes? Is there important variability in how much people value each of the main outcomes? (Not relevant for coverage decisions.)
Do the desirable effects outweigh the undesirable effects?	To what extent do the following considerations influence the balance between desirable and undesirable effects: How much less people value future outcomes compared to outcomes that occur now (their discount rates)? People’s attitudes toward desirable effects (how risk seeking they are). People’s attitudes toward undesirable effects (how risk averse they are).
How large are the resource requirements?	How large is the difference in each item of resource use for which fewer resources are required? How large is the difference in each item of resource use for which more resources are required?
What is the certainty of the evidence of resource requirements?	Have all important items of resource use that may differ between the options being considered been identified? How certain is the evidence of differences in resource use between the options being considered? (See GRADE guidance regarding detailed judgments about the quality of evidence or certainty in estimates.) How certain is the cost of the items of resource use that differ between the options being considered? Is there important variability in the cost of the items of resource use that differ between the options being considered?
Are the net benefits worth the incremental cost?	Judgments regarding each of the six preceding criteria: Is the cost-effectiveness ratio sensitive to one-way sensitivity analyses? Is the cost-effectiveness ratio sensitive to multivariable sensitivity analyses? Is the economic evaluation on which the cost-effectiveness estimate is based reliable? Is the economic evaluation on which the cost-effectiveness estimate is based applicable to the setting(s) of interest?
What would be the impact on health equity?	Are there groups or settings that might be disadvantaged in relation to the problem or options that are considered? Are there plausible reasons for anticipating differences in the relative effectiveness of the option for disadvantaged groups or settings? Are there different baseline conditions across groups or settings that affect the absolute effectiveness of the intervention or the importance of the problem for disadvantaged groups or settings? Are there important considerations that should be made when implementing the intervention in order to ensure that inequities are reduced, if possible, and that they are not increased?
Is the intervention acceptable to key stakeholders?	Are there key stakeholders that would not accept the distribution of the benefits, harms, and costs? Are there key stakeholders that would not accept the costs or undesirable effects in the short term for desirable effects (benefits) in the future? Are there key stakeholders that would not agree with the values attached to the desirable or undesirable effects (because of how they might be affected personally or because of their perceptions of the relative importance of the effects for others)? Would the intervention adversely affect people’s autonomy? Are there key stakeholders that would disapprove of the intervention morally, for reasons other than its effects on people’s autonomy (eg, in relation to ethical principles such as no maleficence, beneficence, and justice)?
Is the intervention feasible to implement?	For decisions other than coverage decisions: Is the intervention or option sustainable? Are there important barriers that are likely to limit the feasibility of implementing the intervention (option) or require consideration when implementing it? For coverage decisions: Is coverage of the intervention sustainable? Is it feasible to ensure appropriate use for approved indications? Is inappropriate use (indications that are not approved) an important concern? Is there capacity to meet increased demand if covered? Are there important legal, bureaucratic, or ethical constraints that make it difficult or impossible to cover the intervention?

## Results

A preliminary search of the literature published between January 1, 1946, and March 1, 2019, was conducted, yielding 617 references and a pilot screening of 100 randomly selected abstracts was performed by three independent reviewers (HS, PV, and CK) using the following inclusion criteria: (1) simulation model use and (2) CRC screening (Figure 1). This resulted in inclusion of 56 of 100 (56%) abstracts after 24 conflicts were resolved through extensive discussion among all authors, leading to revision and clarification of the inclusion criteria as described in the section “Methods”. The revised search yielded 484 references to review. A repeated pilot screening resulted in the inclusion of 8 of 100 (8%) abstracts after 16 conflicts were resolved with minimal discussion. The publication of this article was funded by University of Ottawa Telfer School of Management Research Grant and a Discovery Grant from the Natural Sciences and Engineering Research Council of Canada.

The project was funded in May 2019. Data collection took place from January 2008 to March 2019. Data analysis was completed in November 2019, and are expected to be published in spring 2020.

## Discussion

The purpose of simulation modeling in health care is generally to inform a decision [13,33,36]. The extent to which simulation modeling has fulfilled this purpose has not been assessed. We anticipate that this systematic review will help address this knowledge gap by assessing the contribution simulation modeling has made to informed decision making in an area of health care where it has been frequently used: CRC screening delivery. We will use the GRADE EtD framework to structure our analysis of potential decision impact of the models. This includes the model’s contribution to determining the feasibility of screening, acceptability of proposed screening strategies by stakeholders, and sustainability of screening over the long term. This analysis will help guide researchers by identifying methods in simulation modeling that have been associated with a greater success in decision support, such as mixed methods, participatory simulation model development, and group model building, which has been reported as beneficial in other applications of simulation modeling in health care [37,38]. It will assist decision makers and model users to identify areas where simulation modeling has proven to be useful, such as for identifying resource requirements or conducting cost-benefit analysis.

The dataset search yielded 484 references, which suggests that the body of literature on this topic is fairly robust. We anticipate there will be an adequate number of relevant models to conduct an informative systematic review on this topic.

We foresee several potential limitations to this study. The heterogeneity of articles may make it challenging to evaluate studies using a uniform framework from validation and decision-making criteria. Furthermore, the impact and decision support that a study provide are difficult to quantify and therefore will be subject to both authors’ and reviewers’ bias. We aim to mitigate this by using the GRADE EtD framework and by having reviewers with clinical (HS), health informatics (CK), and simulation (PV) expertise review each included study. Finally, our assessment of model validity will be limited by a lack of validation standards in the literature and reporting by authors on their validation process and outcomes [18].

In conclusion, the proposed systematic review will provide an insight into the contribution and validity of simulation modeling in CRC screening. The results have the potential to inform researchers, health care leaders, and policy makers to develop valid, informative simulation models that will support decision making. This analysis could be expanded to assess the use of simulation modeling in other areas of health care.

## Appendix

Multimedia Appendix 1 PRISMA-P (Preferred Reporting Items for Systematic review and Meta-Analysis Protocols) checklist.

## Abbreviations

CRC: colorectal cancer
GRADE EtD: Grading of Recommendations Assessment, Development and Evaluation Evidence to Decision
ISPOR-SMDM: International Society for Pharmacoeconomics and Outcomes Research-Society for Medical Decision Making
